# Investigating the Genetic Diversity, Population Differentiation and Population Dynamics of *Cycas segmentifida* (Cycadaceae) Endemic to Southwest China by Multiple Molecular Markers

**DOI:** 10.3389/fpls.2017.00839

**Published:** 2017-05-19

**Authors:** Xiuyan Feng, Jian Liu, Yu-Chung Chiang, Xun Gong

**Affiliations:** ^1^Key Laboratory for Plant Diversity and Biogeography of East Asia, Kunming Institute of Botany, Chinese Academy of SciencesKunming, China; ^2^University of Chinese Academy of SciencesBeijing, China; ^3^Department of Biological Sciences, National Sun Yat-sen UniversityKaohsiung, Taiwan; ^4^Yunnan Key Laboratory for Wild Plant Resources, Kunming Institute of Botany, Chinese Academy of SciencesKunming, China

**Keywords:** *Cycas segmentifida*, genetic diversity, genetic structure, population dynamics, conservation

## Abstract

Climate change, species dispersal ability and habitat fragmentation are major factors influencing species distribution and genetic diversity, especially for the range-restricted and threatened taxa. Here, using four sequences of chloroplast DNAs (cpDNAs), three nuclear genes (nDNAs) and 12 nuclear microsatellites (SSRs), we investigated the genetic diversity, genetic structure, divergence time and population dynamics of *Cycas segmentifida* D. Y. Wang and C. Y. Deng, a threatened cycad species endemic to Southwest China. High levels of genetic diversity and genetic differentiation were revealed in *C. segmentifida*. Haplotypes of networks showed two evolutionary units in *C. segmentifida*, with the exception of the nuclear gene *GTP* network. Meanwhile, the UPGMA tree, structure and PCoA analyses suggested that 14 populations of *C. segmentifida* were divided into two clades. There was significant effect of isolation by distance (IBD) in this species. However, this species did not display a significant phylogeographic structure. The divergence time estimation suggested that its haplotypes diverged during the Middle Pleistocene. Additionally, the population dynamics inferred from different DNA sequences analyses were discordant. Bottleneck analysis showed that populations of *C. segmentifida* did not experience any recent bottleneck effect, but rather pointed to a contraction of its effective population size over time. Furthermore, our results suggested that the population BM which held an intact population structure and occupied undisturbed habitat was at the Hardy–Weinberg equilibrium, implying that this population is a free-mating system. These genetic features provide important information for the sustainable management of *C. segmentifida*.

## Introduction

Southwest China is seen as one of the world’s biodiversity hotspots because of its complicated terrain, diversified climates and habitats (Myers et al., 2000). The region possesses an extremely high richness of species and concentrates numerous endemic and endangered plant species (Zhao and Gong, 2015). Climatic oscillations in the Pleistocene led to drastic environmental changes repeatedly, which also substantially influenced the species’ distribution and evolution, even extinction (Hewitt, 1996, 2000, 2004; Comes and Kadereit, 1998). Climate change, dispersal, and habitat fragmentation are considered to be major factors influencing the current distribution and genetic diversity of species (Hewitt, 2004; Broquet and Petit, 2009; Arenas et al., 2012, 2014; Mona et al., 2014). In the Pleistocene, most parts of China were not covered by large ice sheet and had a relatively warm climate (Weaver et al., 1998; Williams et al., 1998). But climatic oscillations during the Pleistocene have also had severe effects on the divergence and population dynamics of many extant species (Hewitt, 1996, 2000, 2004; Avise, 2000). In recent years, there has been an increasing body of literature on the effect of climatic oscillations on species’ divergence and population dynamics (Zhan et al., 2011; Liu et al., 2013; Feng et al., 2014, 2016b; Gong et al., 2015).

As an ancient lineage, cycads are ideal materials with which one can explore how plants have responded to historical climate oscillations. Cycads belong to gymnosperms and are considered the most primitive living seed plants. However, recent research has proposed that a synchronous global rediversification occurred in cycads during the late Miocene (Nagalingum et al., 2011). Although the cycad lineage is ancient, the extant cycad species have evolved recently and are not older than 12 million years based on a fossil-calibrated molecular phylogeny (Nagalingum et al., 2011). They are distributed in tropical and subtropical regions and comprise two families (Cycadaceae, Zamiaceae) with 10 genera (Christenhusz et al., 2011). There is only one cycad genus, *Cycas* (Cycadaceae), in China (Hill et al., 2004). A study combining ancestral area reconstructions with fossil evidence revealed that South China is the origin of *Cycas* (Xiao and Moller, 2015). In South China, *Cycas* species usually grow on low-altitude slopes of ridges and cliffs along river valleys. *Cycas* species in China are all facing potential endangerment challenges due to over-collection because of their edible stem as well as ornamental attributes and the destruction of their habitats for the cultivation of commercial plants. Due to their evolutionary importance, cycads have been studied in many fields, including phylogeny, population genetics, phylogeography and conservation (Chiang et al., 2009; Cibrian-Jaramillo et al., 2010; Zhan et al., 2011; Feng et al., 2014, 2016a,b; Gong et al., 2015; Liu et al., 2015; Xiao and Moller, 2015; Zheng et al., 2016; Yessoufou et al., 2017). However, there are still some *Cycas* species that have not yet been targeted for protection.

For example, *C. segmentifida* D. Y. Wang and C. Y. Deng (Wang and Deng, 1995) draws little attention. It is endemic to Southwest China, and occurs primarily in the valleys of the You River basin of the eastern Yunnan, southwestern Guizhou, and northwestern Guangxi provinces. This species is characterized by its blue–green petiole of young leaves and dichotomous or sometimes forked lateral spine of megasporophyll (Wang and Deng, 1995). After the description of *C. segmentifida*, several new species of *Cycas* in this region were published, based on one or more specific morphological feature (Zhang and Zhong, 1997; Zhang et al., 1997, 1999). These later-described species are morphologically similar to *C. segmentifida*, giving rise to the long-controversial confusion on species classification (Chen and Stevenson, 1999; Wang, 2000; Huang, 2001; Whiteloek, 2002; Ma, 2005). Combining evidence from chloroplast and nuclear DNA sequences, microsatellite analysis, and the geographical distribution, these ambiguous species were delimited and included in *C. segmentifida* (Feng et al., 2016a). *Cycas segmentifida* occupies two types of habitat according to the soil matrix, sand and karst, on which it grows. The populations of the sand type are found under the forest canopy, while the populations of the karst type are scattered on isolated limestone hills with few shrubs. The two types of habitat with obviously different environments lead to morphological differences, such as the length and width of leaflets or acuminate apex of pinnae and shorter carpophylls.

This species is dioecious, allogamous, and insect pollinated. Its pollinators are mainly beetles. As an inland *Cycas* species, *C. segmentifida* is classified into *Cycas* section *Stangerioides* Smitinand which has seeds that are short on thick spongy tissue, always sink in water and contain virulent cycasin, precluding their dispersal by water or animals for long distances (Dehgan and Yuen, 1983; Schneider et al., 2002; Xiao and Moller, 2015). Like other *Cycas* species from sect. *Stangerioides*, its fertile seeds are large and heavy, usually falling and germinating near the mother plant, as found in field survey.

Here, we performed a comprehensive study using four cpDNAs [*psb*A-*trn*H (Shaw et al., 2005), *psb*M-*trn*D (Shaw et al., 2005), *trn*S-*trn*G (Shaw et al., 2005), and *trn*L-*trn*T (Taberlet et al., 1991)], three nDNAs [*GTP*, GTP genes (Salas-Leiva et al., 2014); *PHYP*, phytochrome P gene and *PPRC*, hypothetical protein gene (unpublished)], and 12 microsatellite markers (Cibrian-Jaramillo et al., 2008; Wang et al., 2008; Yang et al., 2008; Li et al., 2009; Zhang et al., 2009, 2010; Ju et al., 2011) to investigate genetic variations, genetic structure, divergence and population dynamics in *C. segmentifida*. We aimed to address the following questions: (1) What is the genetic diversity level of *C. segmentifida*? (2) Are the 14 populations of this species divided into two groups according to the sand and karst habitats? (3) When did the haplotpes of *C. segmentifida* began to diverge, and how did the population dynamics respond to climate fluctuations during historical period?

## Materials and Methods

### Sampling and Genotyping

**FIGURE 1:**
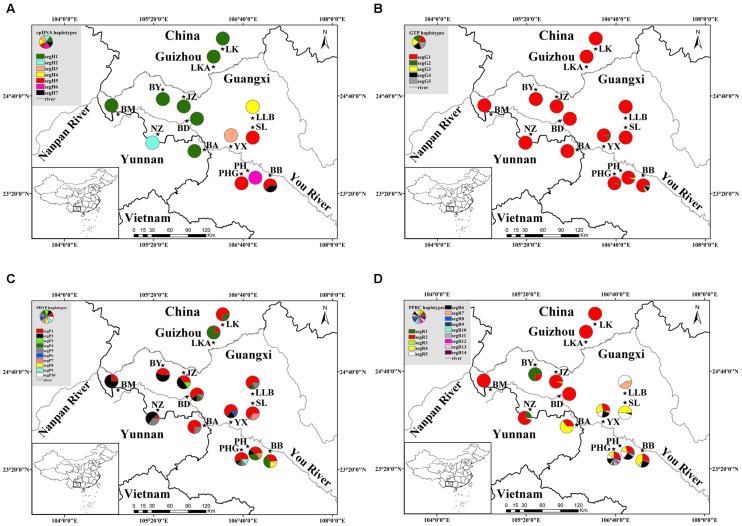
**Geographical distribution of 14 populations of *C. segmentifida* and distribution of its haplotypes detected from cpDNA** **(A)**, *GTP* **(B)**, *PHYP* **(C)**, and *PPRC* **(D)**. Population codes refer to Supplementary Table S1.

A total of 238 individuals were obtained from 14 populations, representing the entire natural distribution area of *C. segmentifida*. Young and healthy leaves were dried in silica gel immediately after collection. Within the 238 samples, 7 to 10 individuals from each population were chosen for chloroplast and nuclear DNA sequencing, while all of the 238 individuals were used for the microsatellite study. Geographical information of the 14 populations and numbers of individuals used in the DNA sequencing and microsatellite analyses are presented in Supplementary Table S1 and **Figure 1**, respectively.

We extracted total DNA using the modified CTAB method (Doyle, 1991) and then sequenced four cpDNA intergenic spacers, *psb*A-*trn*H, *trn*S-*trn*G, *psb*M-*trn*D, and *trn*L-*trn*T, and three nuclear genes, *GTP*, *PHYP*, and *PPRC*, for complete analysis after preliminary screening from universal chloroplast and nuclear primers. PCR amplification procedures were the same as those used in *C. simplicipinna* (Feng et al., 2014). The PCR products were sequenced on an ABI 3770 automated sequencer at Shanghai Major Biological Medicine and Technology Co Ltd in both directions. All sequences were deposited in GenBank with the accession numbers KU240448-KU240465, KU240469-KU240471, KU240474-KU240488, KU240490-KU240491 (Feng et al., 2016a); KT824880-KT824887, KT824890-KT824891 (Feng et al., 2016b); KY292488-KY292501.

Microsatellite loci were screened from nuclear microsatellites which were developed for other *Cycas* plants (Cibrian-Jaramillo et al., 2008; Wang et al., 2008; Yang et al., 2008; Li et al., 2009; Zhang et al., 2009, 2010; Ju et al., 2011). PCR amplification and microsatellite genotyping were conducted with the same protocol as used in *C. simplicipinna* (Feng et al., 2014). Finally, we chose 12 polymorphic microsatellite loci, the same used in our study on species delimitation of the *C. segmentifida* complex (Feng et al., 2016a), for *C. segmentifida*.

### Data Analysis of DNA Sequences

SeqMen was used to edit and assemble sequences. Sequence multiple alignments were subsequently performed in Bioedit, version 7.0.4.1 (Hall, 1999). The four cpDNA regions were combined for a congruency test using PAUP^∗^ 4.0b10 (Swofford, 2003). For three nuclear genes, heterozygous sites were identified by overlapping peaks in chromatograms, and the nuclear sequences were resolved by applying the algorithms of PHASE (Stephens et al., 2001; Stephens and Donnelly, 2003) in the software package DnaSP, version 5.0 (Librado and Rozas, 2009). The combined cpDNA sequences and the phased nuclear sequences were used in analyses that followed. The mapping work was done using the ArcGIS 10.2 software (Esri Inc.).

First, we used DnaSP, version 5.0, to detect recombination in nuclear genes. We calculated indices to measure the level of genetic variation, haplotypes, *Nei’s* nucleotide diversity (*P*i) and haplotype diversity (*H*d). Gene diversity in total populations (*H*_T_), within-population gene diversity (*H*_S_), (Nei, 1973) and two indices of genetic differentiation, *N*_ST_ and *G*_ST_, were also calculated using Permut 1.0 (Pons and Petit, 1996). We compared *G*_ST_ and *N*_ST_ using the *U* test. An estimation of genetic variation that was assigned within and among populations was performed with an analysis of molecular variance (AMOVA) using Arlequin, version 3.11 (Excoffier et al., 2005). The pollen/seed migration ratio (*r*) was calculated using a modified equation: *r* = mp/ms = [(1/*F*_ST_ (n) - 1) - 2(1/*F*_ST_ (c) - 1)]/(1/*F*_ST_ (c) - 1) (Ennos, 1994; Petit et al., 2005), where *F*_ST_ values (rather than *G*_ST_) taken as estimators of population differentiation are derived from AMOVA, mp is the pollen migration rate, ms is the seed migration rate, *F*_ST_ (n) is the nuclear (nDNA) *F*_ST_ and *F*_ST_ (c) is the chloroplast (cpDNA) *F*_ST_. Here, *r*_G_ = mp (*GTP*)/ms; *r*_P_ = mp (*PHYP*)/ms; *r*_R_ = mp (*PPRC*)/ms.

Phylogenetic relationships of haplotypes were constructed using Bayesian methods implemented in MrBayes, version 3.1.2 (Ronquist and Huelsenbeck, 2003), with *Cycas edentata* and *Cycas rumphii* as outgroups. We also used Network, version 4.2.0.1 (Bandelt et al., 1999), to estimate the degree of relatedness among cpDNA and nDNA haplotypes with indels treated as single mutational events.

The evolutionary rates previously estimated for seed plants, 1.01 × 10^-9^ and 5.1–7.0 × 10^-9^ (Graur and Li, 2000) mutations per site per year for synonymous sites, were used to estimate the coalescent time of haplotypes for cpDNA and nDNA, respectively. We used BEAST, version 1.6.1 (Drummond and Rambaut, 2007), to estimate the time of divergence using a strict molecular clock and the HKY model that was determined by MEGA, version 5, based on the Akaike Information Criterion (Tamura et al., 2011). We also used the BEAST program to create a Bayesian Skyline Plot to infer the history demography for *C. segmentifida*. Time of divergence and the mutation rate posterior estimates were obtained by Markov Chain Monte Carlo (MCMC) analysis. The ESS parameter of each process was checked by TRACER, version 1.5 (Rambaut and Drummond, 2009), until a stable value exceeding 200 was reached, suggesting that there was acceptable mixing and sufficient sampling. The subsequent three stable running log files and tree files were combined into pairs. The combined log and tree files were used to construct a Bayesian skyline plot in TRACER, version 1.5. We used DnaSP, version 5.0, to examine a pairwise mismatch distribution and neutrality tests, including Tajima’s *D*, Fu and Li’s *D^∗^* and *F^∗^* and Fu’s *F*_S_ (Fu, 1997), to further investigate population dynamics of the species. The sum-of-squared deviations (SSD) and raggedness index as well as *P*-values were calculated with the software Arlequin, version 3.11 (Excoffier et al., 2005).

### Data Analysis of Microsatellites

Dataset editing and formatting were performed in GenAlEx, version 6.3 (Peakall and Smouse, 2006). Genetic diversity indices, including the number of alleles (*N*_A_), effective number of alleles (*A*_E_), private alleles (*A*_P_), expected heterozygosity (*H*_E_), observed heterozygosity (*H*_O_), information index (*I*), fixation index (*F*) and percentage of polymorphic loci (*PPB*), were calculated using GenAlEx, version 6.3, and POPGENE, version 1.32 (Yeh et al., 1997), with mutual correction. Allelic richness (*A*_R_) was calculated in the software FSTAT, version 1.2 (Goudet, 1995). The differentiation index *F*_ST_ between pairs of populations was computed with Arlequin, version 3.11 (Excoffier et al., 2005). Isolation by distance (IBD) was tested by performing Mantel tests in GenAlEx, version 6.3, on the correlation of genetic distance [*F*_ST_/(1 -*F*_ST_)] with geographic distance for all pairs of populations. Genepop, version 4.1.4 (Rousset, 2008) was used to calculated *F*_ST_/(1 -*F*_ST_). The pollen/seed migration ratio (*r*) was also calculated. Tests for departure from Hardy–Weinberg equilibrium (HWE) were performed in each locus and each population as well as a globally unified population using Genepop, version 4.1.4 (Rousset, 2008).

To gain insight into the population genetic structure of *C. segmentifida*, multiple approaches were used in this study. Initially, the unweighted pair group mean analysis (UPGMA) was performed using TEPGA, version 1.3 (Miller, 1997), with 5,000 permutations. Next, we conducted a Bayesian analysis of population structure with STRUCTURE, version 2.2 (Pritchard et al., 2000). Number of clusters (*K*) was set from 1 to 20, and each *K* run 20 times under 1 × 10^5^ subsequent MCMC samplings and a subsequent burn-in of 1 × 10^5^ iterations. The combination of admixture and correlated-allele frequencies model was used in this analysis. We evaluated the most likely number of groupings using Δ*K* and the log-likelihood value in the program STRUCTURE HARVESTER, version 0.6.8 (Earl and vonHoldt, 2012). Finally, based on Nei’s (1973) genetic distances, an individual-based principal coordinate analysis (PCoA) was conducted using MVSP, version 3.12 (Kovach, 1999).

The effective population sizes of each population were estimated in the program LDNe at three levels of the lowest allele frequency (0.01, 0.02, 0.05) with a 95% confidence interval (Waples and Do, 2008). The bottleneck effect based on different models and methods was tested in BOTTLENECK, version 1.2.02 (Piry et al., 1999), aiming to explore population dynamics. In this analysis, we chose the stepwise mutation model (SMM) and the two-phased model (TPM). Under the two models, the standardized differences test was removed from this study because this test is typically used only when at least 20 polymorphic loci are available. Two other methods (Sign tests and Wilcoxon tests) were applied in the analyses. We also used a mode shift model to test for bottlenecks in each population. These methods implemented in BOTTLENECK are most powerful unless bottlenecks are severe and recent. Moreover, we further investigated a genetic bottleneck using the Garza–Williamson index (GWI, also called *M*-ratio, the ratio of number of alleles to range in allele size) (Garza and Williamson, 2001) which was calculated by Arlequin, version 3.11 (Excoffier et al., 2005). When seven or more loci are analyzed, the GWI is lower than the critical *M*c value of 0.68, a value obtained from bottlenecked populations, which suggests population decline in history (Garza and Williamson, 2001; Excoffier et al., 2005).

## Results

### DNA Sequence Variations

The combined cpDNA had a 3,165 bp consensus length with a significant rate of homogeneity (*P* = 1, >0.5) based on the congruency test, suggesting that there was a high degree of homogeneity among the four cpDNA regions. They contained 22 polymorphic sites and seven haplotypes (segH1–segH7) across the 137 individuals (14 populations, Supplementary Table S1) of *C. segmentifida*. Of these, only two haplotypes segH1 and segH5, were shared by multiple populations, whereas the other five haplotypes were specific to one population (Supplementary Table S2). Detection of recombination in nuclear genes showed that no recombination occurred in *GTP*. In contrast, *PHYP* and *PPRC* had one and two recombination events, respectively. The aligned nuclear genes *GTP*, *PHYP*, and *PPRC* had 561, 930, and 718 bp consensus lengths, include 7, 12, and 16 polymorphic sites, and 5, 10, and 14 haplotypes, respectively. In *GTP*, the haplotype segG1 was the most abundant and predominant in all 14 populations. Haplotypes segG4 and segG5 were private. For *PHYP*, haplotype segP1 was the most frequent and was widely shared by all 14 populations. Haplotypes segP3, segp6, segP9, and segP10 were specific to single population JZ, YX, PHG, and BB. In *PPRC*, segR2 was the most widely distributed haplotype. Information on cpDNA and three nDNA haplotypes and their distribution in populations are shown in **Figure 1** and Supplementary Table S2, respectively.

Genetic diversity indices *H*d and *P*i for each population, summarized in Supplementary Table S2, were highly variable among cpDNA and nDNA. In sum, except for population BB, which displayed both cpDNA nucleotide and haplotype diversity, the remaining 13 populations have very low genetic diversity based on cpDNA data. Three nuclear genes showed variable genetic diversity within 14 populations. The nuclear gene *PHYP* had the highest haplotype diversity in *C. segmentifida*, *PPRC* had the highest nucleotide diversity and *GTP* showed the lowest genetic diversity. Total genetic diversity *H*_T_ was higher than the average intrapopulation diversity *H*s, except for the nuclear gene *GTP*, which had basic equal values (Supplementary Table S3), meaning that there were high levels of genetic differentiation (except *GTP*, *N*_ST_ ranging from 0.282 to 0.996; *G*_ST_ ranging from 0.229 to 0.949). *N*_ST_ was not significantly greater than *G*_ST_ (*P* > 0.05), indicating that *C. segmentifida* shows no correspondence between haplotype similarities and their geographic distribution.

The AMOVA revealed that almost all variation (99.80%) was partitioned among populations based on cpDNA data, whereas higher variations (98.98, 71.38, and 57.41%) were revealed within populations than among populations based on nuclear genes *GTP*, *PHYP*, and *PPRC*, respectively. Except for the gene *GTP*, *F*_ST_ values for cpDNA and the other two nuclear genes ranged from 0.286 to 0.998 (**Table 1**), indicating highly significant genetic differentiation among *C. segmentifida* populations. The pollen/seed migration ratios were calculated as *r*_G_ = 49399, *r*_P_ = 1243.755 and *r*_R_ = 581.086, suggesting that pollen flow was significantly higher than seed flow in *C. segmentifida*.

**Table 1:** Analysis of molecular variance (AMOVA) based on DNA sequences and microsatellites for populations of *C. segmentifida*.

Marker	Source of variation	d.f.	Sum of squares	Variance components	Percentage of variation (%)	*F*_ST_	*r*
cpDNA	Among populations	13	1264.622	9.94365	99.80	0.998^∗∗∗^	
	Within populations	123	2.400	0.01951	0.20		
*GTP*	Among populations	13	0.563	0.00037	1.02	0.010	49399
	Within populations	260	9.379	0.03607	98.98		
*PHYP*	Among populations	13	89.763	0.31306	28.62	0.286^∗∗∗^	1243.755
	Within populations	260	203.014	0.78082	71.38		
*PPRC*	Among populations	13	146.242	0.53799	42.59	0.426^∗∗∗^	581.086
	Within populations	260	188.564	0.72525	57.41		
SSR	Among populations	13	383.854	0.79385	22.89	0.229^∗∗∗^	1678.039
	Within populations	462	1235.367	2.67395	77.11		

*^∗∗∗^*P* < 0.001, most significant difference.*

### Haplotype Relationships and Divergence Times

Bayesian inference revealed the phylogenetic relationships among cpDNA and nDNA haplotypes with *C. edentata* and *C. rumphii* as outgroups. The four phylogenetic trees were strongly supported with high Bayesian probabilities, revealing the monophyly for *C. segmentifida* haplotypes (Supplementary Figure S1). The cpDNA haplotype phylogenetic tree showed that haplotypes segH5 and segH7 grouped together, and the other five haplotypes were clustered into a clade, indicating that they were more closely related than others. In the *GTP* haplotype tree, segG5 was the first diverged haplotype, while the relationship of the remaining four haplotypes, which formed a clade, was not resolved. Two clades were also revealed in the *PHYP* haplotypes Bayesian phylogram, showing that haplotypes segP3, segP4, segP6, and segP9 were clustered into one clade, while the other six haplotypes were clustered into another clade, which revealed a closer relationship among them. Fourteen *PPRC* haplotypes were grouped into three clades, and segR14 was the earliest diverged haplotype in the *PPRC* phylogram. Of the other two clades, one contained haplotypes segR3, segR4, segR6, segR9, segR10, segR12, and segR13, and the other comprised the remaining six haplotypes, indicating that they shared closer relationships.

**FIGURE 2:**
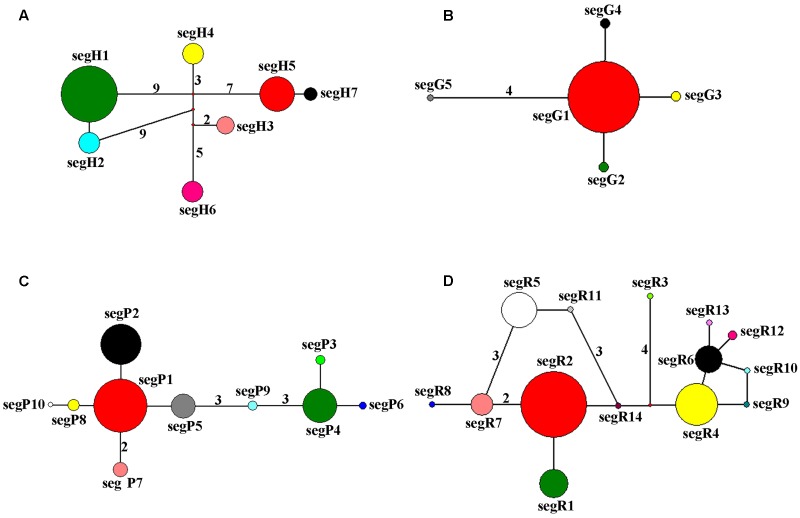
**Network of haplotypes of *C. segmentifida* based on cpDNA (A)**, *GTP* **(B)**, *PHYP* **(C)**, and *PPRC* **(D)**. The numbers on branches indicate mutational steps. Haplotype distribution in 14 populations refers to Supplementary Table S2.

The haplotype network of cpDNA was concordant with the haplotype network of nuclear genes *PHYP* and *PPRC*, which displayed two centrally located nodes representing hypothetical ancestral haplotypes with a higher frequency (**Figures 2A,C,D**). The remaining haplotypes were linked to these central haplotypes by one to nine steps in a star-like network. Absence of some haplotypes caused reticulate evolutionary relationships in networks of cpDNA and *PPRC*. In the network of *GTP* (**Figure 2B**), haplotype segG1 occurred at the highest frequency and was in an internal node location, indicating it may be an ancestral haplotype. The remaining four haplotypes were linked to the central haplotype by one or four steps in a star-like network.

**FIGURE 3:**
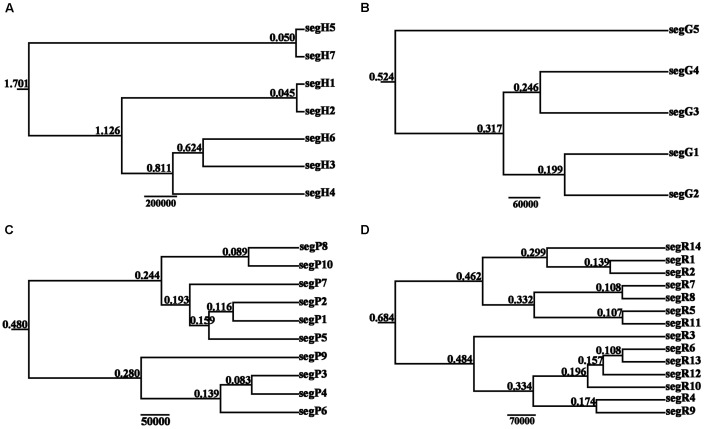
**BEAST-derived trees based on cpDNA (A)** and the nuclear genes *GTP* **(B)**, *PHYP* **(C)**, and *PPRC* **(D)**. The numbers on branches represent divergence time (MYA). Haplotype distribution in 14 populations refers to Supplementary Table S2.

The BEAST-derived trees for the four DNA sequences revealed similar topologies (**Figure 3**). The seven cpDNA haplotypes clustered into two lineages and diverged at approximately 1.701 million years ago (MYA). Of the two lineages, one included five haplotypes with segH1 as the ancestral haplotype and the other included two haplotypes with segH5 as the ancestral haplotype (**Figures 2**, **3A**). For the three nDNA haplotype trees, haplotypes were also divided into two lineages, coalescing at 0.524 MYA (*GTP*), 0.480 MYA (*PHYP*), and 0.684 MYA (*PPRC*), respectively (**Figures 3B–D**). In total, most tip haplotypes in the four BEAST-derived trees diverged recently from their common ancestors. The divergence times estimated from the four DNA sequences entirely implied that haplotypes of *C. segmentifida* diverged in the Middle Pleistocene.

### Neutrality Test, Mismatch Analysis, and the Bayesian Skyline Plot

**FIGURE 4:**
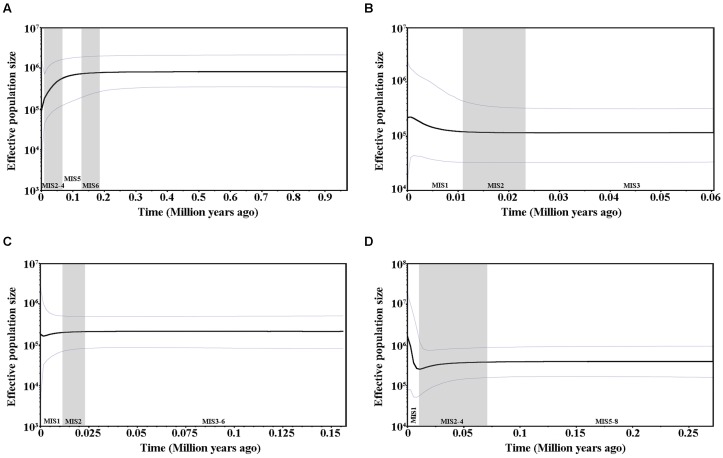
**Bayesian skyline plots based on cpDNA (A)** and the nuclear genes *GTP* **(B)**, *PHYP* **(C)**, and *PPRC* **(D)** for the estimate of fluctuations in effective population size over time. Black line: median estimation; area between gray lines: 95% confidence interval. MIS, Marine Isotope Stage.

Values for Tajima’s *D*, Fu and Li’s *D^∗^*, and *F^∗^*, Fu’s *F*s tests as well as SSD and raggedness statistics are presented in Supplementary Table S4. All of the values were positive and significant for cpDNA and were positive and non-significant for *PHYP*, indicating that *C. segmentifida* had not undergone a recent population expansion. For the nuclear gene *GTP*, except for SSD and raggedness, all values were negative and significant, suggesting the presence of population growth. The nuclear gene *PPRC* showed negative values for Tajima’s *D* as well as for Fu and Li’s *D^∗^* and *F^∗^*, indicating *C. segmentifida* had undergone a recent population expansion. Mismatch distribution analyses displayed multimodal graphs for cpDNA, *PHYP*, and *PPRC*, but a unimodal graph for *GTP* (Supplementary Figure S2). A unimodal curve is indicative of recent population expansion, whereas a multimodal curve is indicative of a population at demographic equilibrium. More detailed evidence of population dynamics came from the Bayesian Skyline Plots. Based on cpDNA data, we found that the population size of *C. segmentifida* remained constant over a long period of time; the population experienced a contraction only recently, i.e., between 0.1 MYA and the present day (**Figure 4A**). During the recent 0.01 million years, rapid population growth and subsequently stabilization were detected in *C. segmentifida* based on the gene *GTP* (**Figure 4B**), while the Bayesian Skyline Plot of the gene *PHYP* (**Figure 4C**) showed this species has had a population contraction since approximately 0.02 MYA, with subsequent slight expansion in demography. In contrast, the Bayesian Skyline Plot of the gene *PPRC* showed that *C. segmentifida* experienced a constant population size over 0.27–0.07 MYA, before undergoing a decline from approximately 0.07 MYA, and then experienced a population expansion from 0.01 MYA to present (**Figure 4D**).

### Nuclear Microsatellite Genotyping

**FIGURE 5:**
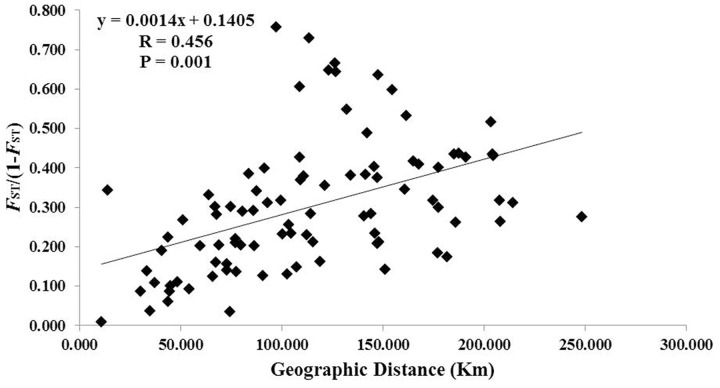
**Figure plot of geographical distance against genetic distance for 14 populations of *C. segmentifida***.

A total of 117 alleles were identified by the 12 microsatellites across the 238 individuals of *C. segmentifida*, and the number of alleles per locus was between 3 (Cy-TaiEST-SSR11) and 44 (cha-estssr01). The locus cha-estssr01 had the highest genetic diversity while Cy-TaiEST-SSR11 had the lowest by comparison of genetic parameters *A*_R_, *N*_A_, *A*_E_, and *I* (Supplementary Table S5). Diversity estimates from 12 microsatellites also varied among populations (**Table 2**), with the highest and lowest measures of diversity consistently found in populations BB and LK, respectively. Fixation indices (*F*) (Wright, 1978) were negative for populations JZ, BM, BD, and LLB, but positive for the other 10 populations, with a mean value *F* = 0.066, indicating outcrossing within those four populations but inbreeding within most populations. This inbreeding resulted in a deficiency of heterozygotes and significant deviations from HWE at most loci and in most populations (Supplementary Table S6). It was noteworthy that population BM is accorded with HWE. Effective population sizes at the lowest allele frequency (= 0.05) are shown in **Table 2**, revealing only three populations, BD, PH, and BB, whose Ne (81.4, 72.3, 71.5) were greater than 50. The AMOVA revealed that more variation (77.11%) was partitioned within populations than between populations, with the significant genetic differentiation coefficient *F*_ST_ = 0.229 (**Table 1**). The pollen/seed migration ratio (*r*) was evaluated as 1678.039, suggesting more pollen flow than seed flow. The correlation between genetic and geographic distances was significant (*P* = 0.001, <0.05), suggesting that *C. segmentifida* has significant effect of IBD (**Figure 5**).

**Table 2:** Genetic diversity within populations of *C. segmentifida*.

Population	*N*_T_	*A*_P_	*A*_R_	*N*_A_	*A*_E_	*I*	*H*_O_	*H*_E_	UHE	*F*	*PPB* (%)	Ne
BY	52	3	2.794	4.333	2.197	0.834	0.325	0.410	0.420	0.156	83.33	8.8
JZ	39	0	2.859	3.250	2.507	0.848	0.450	0.458	0.482	-0.006	83.33	2.6
LK	36	2	2.306	3.000	2.019	0.629	0.263	0.332	0.340	0.133	75.00	23.8
LKA	38	0	2.296	3.167	1.886	0.640	0.258	0.345	0.354	0.266	75.00	16.4
BM	38	2	2.702	3.167	1.833	0.662	0.371	0.364	0.373	-0.044	91.67	22.9
NZ	52	2	2.328	4.333	2.278	0.868	0.360	0.441	0.452	0.123	91.67	36.6
BD	53	1	2.825	4.417	2.697	1.015	0.511	0.513	0.531	-0.026	91.67	81.4
BA	52	3	3.226	4.333	2.150	0.782	0.317	0.376	0.386	0.095	83.33	17.6
YX	41	0	3.123	3.417	2.401	0.855	0.379	0.449	0.485	0.102	75.00	-
LLB	41	0	2.693	3.417	2.283	0.768	0.424	0.401	0.418	-0.087	83.33	-
SL	42	8	2.691	3.500	2.309	0.782	0.351	0.407	0.422	0.112	75.00	38.9
PH	75	2	3.677	6.250	3.650	1.164	0.467	0.517	0.530	0.068	91.67	72.3
PHG	67	6	3.605	5.667	3.698	1.141	0.471	0.522	0.535	0.037	91.67	25.3
BB	72	4	3.877	6.000	4.007	1.252	0.513	0.571	0.586	0.049	91.67	71.5
Mean	57	2.357	2.929	4.161	2.565	0.874	0.390	0.436	0.451	0.066	84.52	34.8

**FIGURE 6:**
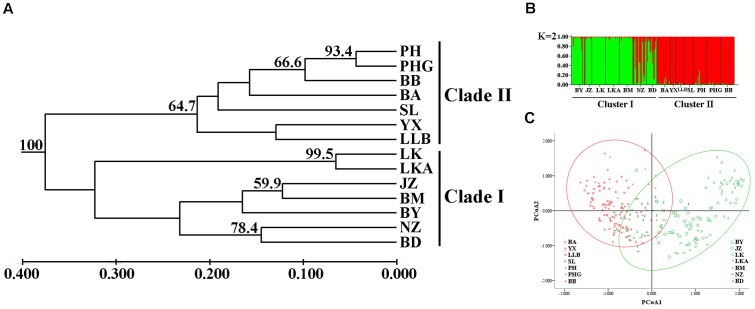
**(A)** An unweighted pair-group method with arithmetic averages (UPGMA) phenogram (numbers on branches indicate bootstrap values from 5,000 replicates), **(B)** Bayesian inference using STRUCTURE (*K* = 2) and **(C)** Principal coordinates analysis (PCoA) of SSR phenotype from 14 populations of 238 individuals of *C. segmentifida*.

The UPGMA clustering dendrogram showed that individuals belonging to populations BY, JZ, LK, LKA, BM, NZ, and BD clustered into one group (Clade I), while the remaining individuals clustered into a second group (Clade II) (**Figure 6A**). Structure (**Figure 6B**) and PcoA (**Figure 6C**) revealed the same results as UPGMA, indicating that 14 populations were grouped into two clusters.

No population had a significant excess of heterozygosity in the two methods under two models in the bottleneck analysis, indicating that 14 populations did not deviate from mutation-drift equilibrium (**Table 3**). Meanwhile, Mode shift model tests revealed that all of the populations were under a normal L-shaped distribution, indicating that *C. segmentifida* had not experienced a severe bottleneck recently. However, Garza-Williamson indices of 14 populations were lower than the critical *M*c value of 0.68 (**Table 3**), indicating that *C. segmentifida* had apparently experienced a population size decline (bottleneck) in history.

**Table 3:** Bottleneck analyses for 14 populations of *C. segmentifida*.

Population	TPM		SMM		Mode shift	Garza–Williamson index
	Sign test	Wilcoxon test	Sign test	Wilcoxon test		
BY	0.404	1.000	0.201	0.233	L	0.378
JZ	0.013ˆ*	0.000ˆ***	0.214	0.008ˆ**	L	0.383
LK	0.020ˆ*	0.012ˆ*	0.020ˆ*	0.054	L	0.371
LKA	0.352	0.151	0.575	0.569	L	0.415
BM	0.183	0.301	0.207	0.677	L	0.404
NZ	0.403	0.117	0.390	0.519	L	0.410
BD	0.399	0.424	0.368	0.677	L	0.444
BA	0.177	0.092	0.613	0.791	L	0.370
YX	0.019ˆ*	0.001ˆ**	0.252	0.034ˆ*	L	0.356
LLB	0.067	0.001ˆ**	0.166	0.011ˆ*	L	0.390
SL	0.046	0.011ˆ*	0.178	0.380	L	0.381
PH	0.411	0.622	0.018ˆ*	0.204	L	0.391
PHG	0.183	0.005ˆ**	0.191	0.519	L	0.377
BB	0.073	0.042ˆ*	0.385	0.850	L	0.375

*P is test for heterozygosity excess, ^∗^P < 0.05, significant difference; ^∗∗^P < 0.01, most significant difference; ^∗∗∗^P < 0.001, most significant difference.*

## Discussion

### High Levels of Genetic Variations and Differentiation

Genetic diversity is the basis for a species’ survival, development, and evolution. Generally, geographical distribution, population number and size, and breeding system all affect genetic diversity in plant species (Hamrick et al., 1992; Hamrick and Godt, 1996; Nybom, 2004). Cycads are dioecious and long-lived plants. They have experienced a long process of evolution (Gao and Thomas, 1989; Axsmith et al., 2003) and should own high genetic diversity. However, a recent research proposed that the extant cycads evolved recently (Nagalingum et al., 2011). In Asia, most *Cycas* species are narrowly distributed and have similar life history traits, such as dioecy, pollination syndromes and longer life span (Wu and Raven, 1999). Comparing the genetic diversity of *C. segmentifida* with other Asian *Cycas* species using the similar molecular markers is reasonable. In contrast, *C. segmentifida* had slightly lower genetic diversity than *C. simplicipinna* (Feng et al., 2014), *C. multipinnata* (Gong et al., 2015), *C. guizhouensis* (Feng et al., 2016b) and *C. dolichophylla* (Zheng et al., 2016), but had higher genetic diversity than *C. debaoensis* (Zhan et al., 2011; Gong and Gong, 2016) and *C. diannanensis* (Liu et al., 2015). *H*_T_ estimated from *C. segmentifida* by four cpDNAs was 0.745, which was higher than 170 plant species’ mean value of *H*_T_ = 0.67 (Petit et al., 2005), implying that *C. segmentifida* had a high level of genetic diversity.

Generally, as an ancient and woody gymnosperm species, cycads are considered to possess high genetic diversity within population and low level of genetic differentiation among populations (Hamrick et al., 1992). However, in this study, cpDNA data demonstrated significant population differentiation (*F*_ST_ = 0.998) (Wright, 1978) within *C. segmentifida*, which was much greater than the average *F*_ST_ value estimated from other seed plants based on maternally inherited markers (mean *F*_ST_ = 0.670) (Petit et al., 2005). In contrast to the significant high value of *F*_ST_ obtained with cpDNA, lower values were derived from nuclear genes, but they still indicated high genetic differentiation within *C. segmentifida* (Wright, 1978). The pollen-to-seed migration ratios (*r*) (Ennos, 1994) illustrated that high pollen flow but limited seed flow among populations is the most likely explanation for the higher genetic differentiation based on cpDNA than nuclear genes. It can also explain the AMOVA results that more genetic variation existed among populations and less within populations based on cpDNA, with the opposite based on nuclear genes and microsatellites. Most of the *C. segmentifida* populations deviated significantly from HWE, together with the fixation indices (*F*) (Wright, 1978) basically greater than zero (**Table 2**), illustrating that *C. segmentifida* populations are notably deficient in heterozygosity and have experienced severe levels of inbreeding. The fact is that its large and heavy seeds germinate and grow near the mother plant, increasing the chances of inbreeding within the species. Consequently, this species presents a high level of genetic differentiation.

### Obvious IBD, Two Genetic Groups, and Middle Pleistocene Divergence

Population genetic structure is mainly affected by some factors such as habitat, differentiation history, and gene flow. Usually, populations cluster according to habitat types. For example, populations of *C. debaoensis* were divided into two groups according to two types of habitats (Zhan et al., 2011). Similarly to *C. debaoensis*, *C. segmentifida* occupied two habitat types (sand and karst); however, it is not clear whether its populations can be differentiated into two different clusters. *N*_ST_ was not significantly greater than *G*_ST_ following *U* tests, which indicated that there was no distinct phylogeographic structure in *C. segmentifida*. However, a significant correlation between nuclear genetic and geographic distance in this species was observed, indicating this species was in accord with IBD. In addition, a clear genetic structure of two genetic groups was detected in *C. segmentifida*, but the two genetic groups did not completely comply with the division of sand and karst habitats. The two central populations (BA and YX, Supplementary Table S1) distributed in the sand habitat were grouped into the karst habitat based on the microsatellite data, perhaps indicating the pollen gene flow occurred between them and the vicinity populations. Our result confirmed higher pollen flow than seed flow in *C. segmentifida*, and this was also demonstrated in other studies (Gong and Gong, 2016). The two populations growed on sand matrix may migrate to the karst habitat and adapt to distinct habitat under the circumstance of ecological selection (Wang et al., 2013).

Parsimony network analysis (except for the nuclear gene *GTP*) suggested that there are two star-like evolutionary units separately dominating two lineages (**Figures 2A,C,D**). The fewer mutational steps indicated a recent rapid divergence. Additionally, the UPGMA, Structure analysis and PCoA clustering of microsatellite data showed the 14 populations comprise two clades (I and II) (**Figure 6A**), and with similar patterns in haplotype distributions of the gene *PPRC* (**Figure 1D**), but had a little conflict with cpDNA haplotype distributions (**Figure 1A**). The population BA situated at the border of the two clades geographically clustered into Clade II based on microsatellites, while clustered into another group with the seven populations belonging to Clade I based on cpDNA haplotype distributions. This little conflictual relationship may reflect ongoing pollen-dispersed gene flow among nearby populations, insufficient lineage sorting at nuclear loci or shared ancestry due to recent species divergence. Meanwhile, the BEAST-derived trees also revealed that haplotypes were separated into two groups with an ancestral haplotype at the inner node of each group, further indicating a recent divergence.

Based on the BEAST-derived trees, the estimated divergence times within *C. segmentifida* fell within the Middle Pleistocene, further certifying that this lineage was the product of a recent rapid divergence, which is consistent with the former conclusion that the extant cycad species have been evolving recently (Nagalingum et al., 2011). Climatic oscillations in the Pleistocene are often regarded as a major factor shaping genetic diversity of many extant species (Hewitt, 1996, 2000, 2004; Avise, 2000; Yessoufou et al., 2014). Although the Pleistocene ice sheet was evident during major glaciations in Europe (Taberlet et al., 1998), a relatively mild unglaciated Quaternary climate occurred in China, except at higher elevations (Weaver et al., 1998). In China, the lower slopes or valleys were not affected during the cooler periods (Qu et al., 2011). Therefore, we propose that the relatively mild Pleistocene climate in Southwest China contributed to the survival and divergence of *C. segmentifida*.

### Different DNA Markers Reveal Different Patterns of Population Dynamics

In this study, cpDNA, three nuclear genes and microsatellites revealed inconsistent population dynamics for *C. segmentifida*. Based on cpDNA data, results of neutrality test and mismatch analysis suggested that *C. segmentifida* has not recently experienced population expansion events, which may be caused by long-term geographical isolation or geographical division. However, the Bayesian Skyline Plot revealed that *C. segmentifida* was in a stable state for a long time, until approximately 0.1 MYA when its population started shrinking. Population dynamics revealed by three nuclear genes were also inconsistent in this study. Nuclear gene *GTP* revealed that *C. segmentifida* experienced a recent population expansion. Our results from the nuclear gene *PHYP* showed that *C. segmentifida* has had a recent population contraction. For the nuclear gene *PPRC*, with the exception of the result of mismatch analysis, the neutrality test and Bayesian Skyline Plot revealed *C. segmentifida* had a recent population expansion. From the results of DNA sequences, two nuclear genes, *GTP* and *PPRC*, showed that *C. segmentifida* experienced population expansion in Marine Isotope Stages 1 (MIS1) (Ogg et al., 2008), namely, the postglacial period while cpDNA and nuclear gene *PHYP* showed population contraction in MIS1. In addition, BOTTLENECK analysis based on microsatellites showed that populations of *C. segmentifida* have not experienced a recent bottleneck event, but GWI estimation revealed these populations experienced a historical reduction in population size. Normally, different genes and markers are subjected to different selective pressures, which may generate the above inconsistency.

Distinct population dynamics were detected in different plant taxa as a result of glacial and interglacial climate oscillations. Gymnosperm species, such as *C. debaoensis* (Zhan et al., 2011), *C. simplicipinna* (Feng et al., 2014) and *C. multipinnata* (Gong et al., 2015), have experienced population contractions during the most recent glacial period, while *Taxus wallichiana* (Liu et al., 2013), *Cycas revoluta* and *Cycas taitungensis* (Chiang et al., 2009) have experienced population expansions. Improved knowledge about the history dynamic of species will help us to predict how they will react to environmental fluctuations in the future and to propose conservation strategies for species (Porretta et al., 2007; Lyons et al., 2012; Wei et al., 2013).

### Conservation Suggestion

In the wild, *C. segmentifida* is distributed only in the boundaries of Yunnan, Guizhou, and Guangxi provinces. The destruction of its habitats for planting commercial or medicinal crops and massive illegal digging of this species for trading or ornaments has led to a sharp reduction of its population size. Delaying in conservation decision-making would give rise to the danger of extinction for this species. Conservation management decisions must be made rapidly to prevent this endangered species from extinction.

The objective of conservation of threatened species is to maintain their contribution to overall genetic diversity (Montalvo et al., 1997). This study detected a relatively higher level of genetic diversity in *C. segmentifida* than in some other *Cycas* species. If an effective population size is greater than 100, it can prevent inbreeding depression. However, the estimated Ne in most populations of *C. segmentifida* was less than 50, and in many cases less than 10, such as populations BY and JZ (**Table 2**). According to the wild field investigation, we found that the habitat of population BM is not destroyed, the regeneration ability of the population is very strong, it has a high population density, and different ages of individuals exist in the population. The HWE test showed that the population BM was in HWE, indicating that it is a free-mating group. It was habitat destruction and massive illegal digging of *C. segmentifida* that jeopardized the existing populations of this species and its effective population size. Therefore, we suggest that protection zones or plots in the distribution areas of *C. segmentifida* be established to protect the habitat for this species. In this study, two genetic clusters were detected in *C. segmentifida*. We proposed that these two genetic clusters could be managed as two evolutionary units and should be given the highest priority protection. The divergence of *C. segmentifida* and network of its haplotypes indicated that this lineage recently evolved rapidly. We suggest protecting the species in its natural habitat (*in situ*). Alternatively, *ex situ* conservation such as seeds or seedling collection from distinct populations that possess pivotal genetic components and reintroduction are needed. Meanwhile, wild introgression from other *Cycas* species should be avoided. In addition, more efforts should be made to raise local farmers’ conservation awareness and a prohibition on deforestation in *Cycas* distribution areas should be implemented.

## Author Contributions

XF was in charge of finishing the molecular genetic studies, performing the data analysis and writing the manuscript. JL contributed to materials collection, DNA extraction, and manuscript revision. XG and Y-CC designed the study, collected research materials and drafted the manuscript. All authors read and approved the final manuscript.

## Conflict of Interest Statement

The authors declare that the research was conducted in the absence of any commercial or financial relationships that could be construed as a potential conflict of interest.

## References

[B1] Arenas M. Mona S. Trochet A. Sramkova H. A. Currat M. Ray N. (2014). “The scaling of genetic diversity in a changing and fragmented world,” in *Ecology and Biodiversity Conservation*, eds Henle K. Potts S. G. Kunin W. E. Matsinos Y. G. Similä J. Pantis J. D. (Sofia: Pensoft Publishers), 55–60.

[B2] Arenas M. Ray N. Currat M. Excoffier L. (2012). Consequences of range contractions and range shifts on molecular diversity. *Mol. Biol. Evol.* 29 207–218. 10.1093/molbev/msr187 21778191

[B3] Avise J. C. (2000). *Phylogeography: The History and Formation of Species.* Cambridge: Harvard University Press.

[B4] Axsmith B. J. Serbet R. Krings M. Taylor T. N. Taylor E. L. Mamay S. H. (2003). The enigmatic paleozoic plants spermopteris and phasmatocycas reconsidered. *Am. J. Bot.* 90 1585–1595. 10.3732/ajb.90.11.1585 21653333

[B5] Bandelt H. J. Forster P. Röhl A. (1999). Median-joining networks for inferring intraspecific phylogenies. *Mol. Biol. Evol.* 16 37–48. 10.1093/oxfordjournals.molbev.a026036 10331250

[B6] Broquet T. Petit E. J. (2009). Molecular estimation of dispersal for ecology and population genetics. *Annu. Rev. Ecol. Evol. Syst.* 40 193–216. 10.1146/annurev.ecolsys.110308.120324

[B7] Chen J. R. Stevenson D. W. (1999). “Cycadaceae,” in *Flora of China*, eds Wu Z. Y. Raven P. H. (Beijing: Science Press and Missouri Botanieal Garden Press), 1–7.

[B8] Chiang Y. C. Hung K. H. Moore S. J. Ge X. J. Huang S. Hsu T. W. (2009). Paraphyly of organelle DNAs in *Cycas* Sect. Asiorientales due to ancient ancestral polymorphisms. *BMC Evol. Biol.* 9:161 10.1186/1471-2148-9-161 PMC3224665 19589178

[B9] Christenhusz M. Reveal J. Farjon A. Gardner M. F. Mill R. R. Chase M. W. (2011). A new classification and linear sequence of extant gymnosperms. *Phytotaxa* 19 55–70. 10.11646/phytotaxa.19.1.3

[B10] Cibrian-Jaramillo A. Daly A. C. Brenner E. Desalle R. Marler T. E. (2010). When North and South don’t mix: genetic connectivity of a recently endangered oceanic cycad, *Cycas micronesica*, in Guam using EST-microsatellites. *Mol. Ecol.* 19 2364–2379. 10.1111/j.1365-294X.2010.04638.x 20497328

[B11] Cibrian-Jaramillo A. Marler T. E. DeSalle R. Brenner E. D. (2008). Development of EST-microsatellites from the cycad *Cycas rumphii*, and their use in the recently endangered *Cycas micronesica*. *Conserv. Genet.* 9 1051–1054. 10.1007/s10592-007-9447-3

[B12] Comes H. P. Kadereit J. W. (1998). The effect of quaternary climatic changes on plant distribution and evolution. *Trends Plant Sci.* 3 432–438. 10.1016/s1360-1385(98)01327-2

[B13] Dehgan B. Yuen C. (1983). Seed morphology in relation to dispersal, evolution, and propagation of *Cycas* L. *Bot. Gazette* 144 412–418. 10.1086/337391

[B14] Doyle J. (1991). “DNA protocols for plants-CTAB total DNA isolation,” in *Molecular Techniques in Taxonomy*, eds Hewitt G. M. Johnson A. (Berlin: Springer), 283–293.

[B15] Drummond A. J. Rambaut A. (2007). BEAST: bayesian evolutionary analysis by sampling trees. *BMC Evol. Biol.* 7:214 10.1186/1471-2148-7-214 PMC2247476 17996036

[B16] Earl D. A. vonHoldt B. M. (2012). Structure Harvester: a website and program for visualizing structure output and implementing the evanno method. *Conserv. Genet. Resour.* 4 359–361. 10.1007/s12686-011-9548-7

[B17] Ennos R. A. (1994). Estimating the relative rates of pollen and seed migration among plant populations. *Heredity* 72 250–259. 10.1038/hdy.1994.35

[B18] Excoffier L. Laval G. Schneider S. (2005). Arlequin (version 3.0): an integrated software package for population genetics data analysis. *Evol. Bioinform.* 1 47–50. PMC2658868 19325852

[B19] Feng X. Y. Liu J. Gong X. (2016a). Species delimitation of the *Cycas segmentifida* complex (*Cycadaceae*) resolved by phylogenetic and distance analyses of molecular data. *Front. Plant Sci.* 7:134 10.3389/fpls.2016.00134 PMC4753401 26913044

[B20] Feng X. Y. Wang Y. H. Gong X. (2014). Genetic diversity, genetic structure and demographic history of *Cycas simplicipinna* (Cycadaceae) assessed by DNA sequences and SSR markers. *BMC Plant Biol.* 14:187 10.1186/1471-2229-14-187 PMC4114127 25016306

[B21] Feng X. Y. Zheng Y. Gong X. (2016b). Middle-upper pleistocene climate changes shaped the divergence and demography of *Cycas guizhouensis* (Cycadaceae): evidence from DNA sequences and microsatellite markers. *Sci. Rep.* 6:27368 10.1038/srep27368 PMC4895228 27270859

[B22] Fu Y. X. (1997). Statistical tests of neutrality of mutations against population growth, hitchhiking and background selection. *Genetics* 147 915–925. 9335623 10.1093/genetics/147.2.915 PMC1208208

[B23] Gao Z. F. Thomas B. A. (1989). A review of fossil cycad megasporophylls, with new evidence of crossozamia pomel and its associated leaves from the Lower Permian of Taiyuan, China. *Rev. Palaeobot. Palynol.* 60 205–223. 10.1016/0034-6667(89)90044-4

[B24] Garza J. C. Williamson E. G. (2001). Detection of reduction in population size using data from microsatellite loci. *Mol. Ecol.* 10 305–318. 10.1046/j.1365-294X.2001.01190.x 11298947

[B25] Gong Y. Q. Gong X. (2016). Pollen-mediated gene flow promotes low nuclear genetic differentiation among populations of *Cycas debaoensis* (Cycadaceae). *Tree Genet. Genom.* 12:93 10.1007/s11295-016-1051-6

[B26] Gong Y. Q. Zhan Q. Q. Nguyen K. S. Nguyen H. T. Wang Y. H. Gong X. (2015). The historical demography and genetic variation of the endangered *Cycas multipinnata* (*Cycadaceae*) in the red river region, examined by chloroplast DNA sequences and microsatellite markers. *PLoS ONE* 10:e0117719 10.1371/journal.pone.0117719 PMC4331093 25689828

[B27] Goudet J. (1995). FSTAT (version 1.2): a computer program to calculate F-statistics. *J. Heredity* 86 485–486. 10.1093/oxfordjournals.jhered.a111627

[B28] Graur D. Li W. H. (2000). *Fundamentals of Molecular Evolution.* Sunderland: Sinauer Associates.

[B29] Hall T. A. (1999). BioEdit: a user-friendly biological sequence alignment editor and analysis program for Windows 95/98/NT. *Nucleic Acids Symp. Ser.* 41 95–98.

[B30] Hamrick J. L. Godt M. J. W. (1996). “Conservation genetics of endemic plant species,” in *Conservation Genetics*, eds Avise J. C. Hamrick J. L. (New York, NY: Springer), 281–304.

[B31] Hamrick J. L. Godt M. J. W. Sherman-Broyles S. L. (1992). Factors influencing levels of genetic diversity in woody plant species. *New Forests* 6 95–124. 10.1007/978-94-011-2815-5_7

[B32] Hewitt G. (2000). The genetic legacy of the Quaternary ice ages. *Nature* 405 907–913. 10.1038/35016000 10879524

[B33] Hewitt G. M. (1996). Some genetic consequences of ice ages, and their role in divergence and speciation. *Biol. J. Linn. Soc.* 58 247–276. 10.1111/j.1095-8312.1996.tb01434.x

[B34] Hewitt G. M. (2004). Genetic consequences of climatic oscillations in the Quaternary. *Philos. Trans. R. Soc. Lon. B Biol. Sci.* 359 183–195. 10.1098/rstb.2003.1388 15101575 PMC1693318

[B35] Hill K. D. Stevenson D. W. Osborne R. (2004). The world list of cycads. *Bot. Rev.* 70 274–298. 10.1663/0006-81012004070

[B36] Huang Y. Y. (2001). *The System Classification and Evolution of Cycadaceae in China.* Beijing: Meteorological Press.

[B37] Ju L. P. Kuo C. C. Chao Y. S. Cheng Y. P. Gong X. Chiang Y. C. (2011). Microsatellite primers in the native perennial cycad *Cycas taitungensis* (Cycadaceae). *Am. J. Bot.* 98 e84–e86. 10.3732/ajb.1000504 21613154

[B38] Kovach W. L. (1999). *MVSP-a Multivariate Statistical Package for Windows, ver. 3.1. Pentraeth.* Wales: Kovach Computing Services.

[B39] Li L. Wang Z. F. Jian S. G. Zhu P. Zhang M. Ye W. H. (2009). Isolation and characterization of microsatellite loci in endangered *Cycas changjiangensis* (Cycadaceae). *Conser. Genet.* 10 793–795. 10.1007/s10592-008-9664-4

[B40] Librado P. Rozas J. (2009). DnaSP v5: a software for comprehensive analysis of DNA polymorphism data. *Bioinformatics* 25 1451–1452. 10.1093/bioinformatics/btp187 19346325

[B41] Liu J. Moller M. Provan J. Gao L. M. Poudel R. C. Li D. Z. (2013). Geological and ecological factors drive cryptic speciation of yews in a biodiversity hotspot. *New Phytol.* 199 1093–1108. 10.1111/nph.12336 23718262

[B42] Liu J. Zhou W. Gong X. (2015). Species delimitation, genetic diversity and population historical dynamics of *Cycas diannanensis* (Cycadaceae) occurring sympatrically in the Red River region of China. *Front. Plant Sci.* 6:696 10.3389/fpls.2015.00696 PMC4562272 26442013

[B43] Lyons J. I. Pierce A. A. Barribeau S. M. Sternberg E. D. Mongue A. J. Roode D. (2012). Lack of genetic differentiation between monarch butterflies with divergent migration destinations. *Mol. Ecol.* 21 3433–3444. 10.1111/j.1365-294X.2012.05613.x 22574833

[B44] Ma Y. (2005). *Studies on Classification and Genetic Diversity of Cycas segmentifida Complex in Baise of Guangxi, China.* Nanning: Guangxi University.

[B45] Miller M. P. (1997). Tools for population genetic analyses (TFPGA) 1.3*:* a Windows program for the analysis of allozyme and molecular population genetic data. *Comput. Softw. Distribut. Author* 4:157.

[B46] Mona S. Ray N. Arenas M. Excoffier L. (2014). Genetic consequences of habitat fragmentation during a range expansion. *Heredity* 112 291–299. 10.1038/hdy.2013.105 24149654 PMC3931167

[B47] Montalvo A. M. Williams S. L. Rice K. J. Buchmann S. L. Cory C. Handel S. N. (1997). Restoration biology: a population biology perspective. *Restor. Ecol.* 5 277–290. 10.1046/j.1526-100X.1997.00542.x

[B48] Myers N. Mittermeier R. A. Mittermeier C. G. Da Fonseca G. A. B. Kent J. (2000). Biodiversity hotspots for conservation priorities. *Nature* 403 853–858. 10.1038/35002501 10706275

[B49] Nagalingum N. S. Marshall C. R. Quental T. B. Rai H. S. Little D. P. Mathews S. (2011). Recent synchronous radiation of a living fossil. *Science* 334 796–799. 10.1126/science.1209926 22021670

[B50] Nei M. (1973). Analysis of gene diversity in subdivided populations. *Proc. Natl. Acad. Sci. U.S.A.* 70 3321–3323. 10.1073/pnas.70.12.3321 4519626 PMC427228

[B51] Nybom H. (2004). Comparison of different nuclear DNA markers for estimating intraspecific genetic diversity in plants. *Mol. Ecol.* 13 1143–1155. 10.1111/j.1365-294X.2004.02141.x 15078452

[B52] Ogg J. G. Ogg G. Gradstein F. M. (2008). *The Concise Geologic Time Scale.* New York, NY: Cambridge University Press.

[B53] Peakall R. Smouse P. E. (2006). GENALEX 6: genetic analysis in Excel. Population genetic software for teaching and research. *Mol. Ecol. Notes* 6 288–295. 10.1111/j.1471-8286.2005.01155.x PMC3463245 22820204

[B54] Petit R. J. Duminil J. Fineschi S. Hampe A. Salvini D. Vendramin G. G. (2005). Invited review: comparative organization of chloroplast, mitochondrial and nuclear diversity in plant populations. *Mol. Ecol.* 14 689–701. 10.1111/j.1365-294X.2004.02410.x 15723661

[B55] Piry S. Luikart G. Cornuet J. M. (1999). BOTTLENECK: a computer program for detecting recent reductions in the effective size using allele frequency data. *J. Heredity* 90 502–503. 10.1093/jhered/90.4.502

[B56] Pons O. Petit R. J. (1996). Measuring and testing genetic differentiation with ordered versus unordered alleles. *Genetics* 144 1237–1245. 8913764 10.1093/genetics/144.3.1237 PMC1207615

[B57] Porretta D. Canestrelli D. Bellini R. Celli G. Urbanelli S. (2007). Improving insect pest management through population genetic data: a case study of the mosquito *Ochlerotatus caspius* (Pallas). *J. Appl. Ecol.* 44 682–691. 10.1111/j.1365-2664.2007.01301.x

[B58] Pritchard J. K. Stephens M. Donnelly P. (2000). Inference of population structure using multilocus genotype data. *Genetics* 155 945–959. 10835412 10.1093/genetics/155.2.945 PMC1461096

[B59] Qu Y. H. Luo X. Zhang R. Y. Song G. Zou F. S. Lei F. M. (2011). Lineage diversification and historical demography of a montane bird *Garrulax* elliotii - implications for the Pleistocene evolutionary history of the eastern Himalayas. *BMC Evol. Biol.* 11:174 10.1186/1471-2148-11-174 PMC3150279 21689460

[B60] Rambaut A. Drummond A. J. (2009). *TRACER: MCMC Trace Analysis Tool Version v1.5.0*. Oxford: University of Oxford Available at: http://tree.bio.ed.ac.uk/software/tracer/">

[B61] Ronquist F. Huelsenbeck J. P. (2003). MrBayes 3: bayesian phylogenetic inference under mixed models. *Bioinformatics* 19 1572–1574. 10.1093/bioinformatics/btg180 12912839

[B62] Rousset F. (2008). GENEPOP’007: a complete re-implementation of the GENEPOP software for Windows and Linux. *Mol. Ecol. Resour.* 8 103–106. 10.1111/j.1471-8286.2007.01931.x 21585727

[B63] Salas-Leiva D. E. Meerow A. W. Francisco-Ortega J. Calonje M. Griffith M. P. Stevenson D. W. (2014). Conserved genetic regions across angiosperms as tools to develop single-copy nuclear markers in gymnosperms: an example using cycads. *Mol. Ecol. Resour.* 14 831–845. 10.1111/1755-0998.12228 24444413

[B64] Schneider D. Wink M. Sporer F. Lounibos P. (2002). Cycads: their evolution, toxins, herbivores and insect pollinators. *Naturwissenschaften* 89 281–294. 10.1007/s00114-002-0330-2 12216856

[B65] Shaw J. Lickey E. B. Beck J. T. Farmer S. B. Liu W. S. Miller J. (2005). The tortoise and the hare II: relative utility of 21 noncoding chloroplast DNA sequences for phylogenetic analysis. *Am. J. Bot.* 92 142–166. 10.3732/ajb.92.1.142 21652394

[B66] Stephens M. Donnelly P. (2003). A comparison of bayesian methods for haplotype reconstruction from population genotype data. *Am. J. Hum. Genet.* 73 1162–1169. 10.1086/379378 14574645 PMC1180495

[B67] Stephens M. Smith N. J. Donnelly P. (2001). A new statistical method for haplotype reconstruction from population data. *Am. J. Hum. Genet.* 68 978–989. 10.1086/319501 11254454 PMC1275651

[B68] Swofford D. L. (2003). *PAUP^∗^: Phylogenetic Analysis Using Parsimony (^∗^ and Other Methods). Version* 4 Sunderland: Sinauer Associates.

[B69] Taberlet P. Fumagalli L. Wust-Saucy A. G. Cosson J. F. (1998). Comparative phylogeography and postglacial colonization routes in Europe. *Mol. Ecol.* 7 453–464. 10.1046/j.1365-294x.1998.00289.x 9628000

[B70] Taberlet P. Gielly L. Pautou G. Bouvet J. (1991). Universal primers for amplification of three non-coding regions of chloroplast DNA. *Plant Mol. Biol.* 17 1105–1109. 10.1007/BF00037152 1932684

[B71] Tamura K. Peterson D. Peterson N. Stecher G. Nei M. Kumar S. (2011). MEGA5: molecular evolutionary genetics analysis using maximum likelihood, evolutionary distance, and maximum parsimony methods. *Mol. Biol. Evol.* 28 2731–2739. 10.1093/molbev/msr121 21546353 PMC3203626

[B72] Wang B. S. Mao J. F. Zhao W. Wang X. R. (2013). Impact of geography and climate on the genetic differentiation of the subtropical pine *Pinus yunnanensis*. *PLoS ONE* 8:e67345 10.1371/journal.pone.0067345 PMC3693954 23840668

[B73] Wang D. Y. (2000). *Morphological Structure, System Classification and Evolutionary Research of Cycadaceae.* Nanjing: Nanjing Forestry University.

[B74] Wang D. Y. Deng C. Y. (1995). *Cycas segmentifida*, a new species from China. *Cycad Soc. S. Afr.* 43 11–14.

[B75] Wang Z. F. Ye W. H. Cao H. L. Li Z. C. Peng S. L. (2008). Identification and characterization of EST-SSRs and cpSSRs in endangered *Cycas hainanensis*. *Conserv. Genet.* 9 1079–1081. 10.1007/s10592-007-9461-5

[B76] Waples R. S. Do C. (2008). LDNE: a program for estimating effective population size from data on linkage disequilibrium. *Mol. Ecol. Resour.* 8 753–756. 10.1111/j.1755-0998.2007.02061.x 21585883

[B77] Weaver A. J. Eby M. Fanning A. F. Wiebe E. C. (1998). Simulated influence of carbon dioxide, orbital forcing and ice sheets on the climate of the last glacial maximum. *Nature* 394 847–853. 10.1038/29695

[B78] Wei S. J. Shi B. C. Gong Y. J. Jin G. H. Chen X. X. Meng X. F. (2013). Genetic structure and demographic history reveal migration of the diamondback moth *Plutella xylostella* (*Lepidoptera*: *Plutellidae*) from the southern to northern regions of China. *PLoS ONE* 8:e59654 10.1371/journal.pone.0059654 PMC3614937 23565158

[B79] Whiteloek L. M. (2002). *The Cycads.* Portland: Timber Press.

[B80] Williams M. A. J. Dunkerley D. L. De Deckker P. Kershaw A. P. Stokes T. (1998). *Quaternary Environments.* London: Arnold Press.

[B81] Wright S. (1978). *Evolution and the Genetics of Populations: Variability Within and Among Natural Populations*, Vol. 4 Chicago, IL: University of Chicago Press

[B82] Wu Z. Y. Raven P. H. (1999). *Flora of China. (Cycadaceae through Fagaceae)*, Vol. 4 Beijing: Science Press.

[B83] Xiao L. Q. Moller M. (2015). Nuclear ribosomal ITS functional paralogs resolve the phylogenetic relationships of a late-miocene radiation cycad *Cycas* (Cycadaceae). *PLoS ONE* 10:e0117971 10.1371/journal.pone.0117971 PMC4311995 25635842

[B84] Yang Y. Li Y. Li L. F. Ge X. J. Gong X. (2008). Isolation and characterization of microsatellite markers for *Cycas debaoensis* YC Zhong et CJ Chen (Cycadaceae). *Mol. Ecol. Resour.* 8 913–915. 10.1111/j.1755-0998.2008.02114.x 21585928

[B85] Yeh F. C. Yang R. C. Boyle T. B. J. Ye Z. H. Mao J. X. (1997). *POPGENE, The User-friendly Shareware for Population Genetic Analysis. Molecular Biology and Biotechnology Centre.* Edmonton, AB: University of Alberta, 10.

[B86] Yessoufou K. Bamigboye S. O. Daru B. H. Bank M. (2014). Evidence of constant diversification punctuated by a mass extinction in the African cycads. *Ecol. Evol.* 4 50–58. 10.1002/ece3.880 24455160 PMC3894887

[B87] Yessoufou K. Daru B. H. Tafirei R. Elansary H. O. Rampedi I. (2017). Integrating biogeography, threat and evolutionary data to explore extinction crisis in the taxonomic group of cycads. *Ecol. Evol.* 7 2735–2746. 10.1002/ece3.2660 28428864 PMC5395460

[B88] Zhan Q. Q. Wang J. F. Gong X. Peng H. (2011). Patterns of chloroplast DNA variation in *Cycas debaoensis* (Cycadaceae): conservation implications. *Conserv. Gen.* 12 959–970. 10.1007/s10592-011-0198-9

[B89] Zhang F. M. Su T. Yang Y. Zhai Y. H. Ji Y. H. Chen S. T. (2010). Development of seven novel EST-SSR markers from Cycas panzhihuaensis (Cycadaceae). *Am. J. Bot.* 97 e159–e161. 10.3732/ajb.1000377 21616839

[B90] Zhang H. D. Zhong Y. C. (1997). New cycas from guangxi. *Acta Sci. Naturalium Universitatis Sunyatseni* 36 67–71. 10.11646/zootaxa.3986.3.1

[B91] Zhang H. D. Zhong Y. C. Huang Y. Y. Zheng H. X. Lu Z. F. (1997). Additions to the cycadaceous flora of China. *Acta Sci. Nat. Univ. Sunyatseni* 37 6–8.

[B92] Zhang H. D. Zhong Y. C. Lu Z. F. (1999). A new species of *Cycas* from Guangxi. *Acta Sci. Nat. Univ. Sunyatseni* 38 121–122. 10.11646/zootaxa.3986.3.1

[B93] Zhang M. Wang Z. F. Jian S. G. Ye W. H. Cao H. L. Zhu P. (2009). Isolation and characterization of microsatellite markers for *Cycas hainanensis* CJ Chen (Cycadaceae). *Conserv. Genet.* 10 1175–1176. 10.1007/s10592-008-9737-4

[B94] Zhao Y. J. Gong X. (2015). Diversity and conservation of plant species in dry valleys, southwest China. *Biodiver. Conser.* 24 2611–2623. 10.1007/s10531-015-0952-2

[B95] Zheng Y. Liu J. Gong X. (2016). Tectonic and climatic impacts on the biota within the Red River Fault, evidence from phylogeography of *Cycas dolichophylla* (Cycadaceae). *Sci. Rep.* 6:33540 10.1038/srep33540 PMC5024324 27629063

