# Supplementary material for: Investigating the Genetic Diversity, Population Differentiation and Population Dynamics of Cycas segmentifida (Cycadaceae) Endemic to Southwest China by Multiple Molecular Markers
Source: Front Plant Sci. 2017 May 19;8:839. doi: 10.3389/fpls.2017.00839 (PMC5437697; doi:10.3389/fpls.2017.00839)
Supplement: Supplementary file 1 [file Presentation_1.PDF]

Supplementary Material

Investigating the genetic diversity, population differentiation and population dynamics of *Cycas segmentifida* (Cycadaceae) endemic to Southwest China by multiple molecular markers

Xiuyan Feng[1,2], Jian Liu[1,2], Yu-Chung Chiang[3*], and Xun Gong[1*]

*** Correspondence:** Corresponding Authors: **Xun Gong:** gongxun@mail.kib.ac.cn;

Yu-Chung Chiang : yuchung@mail.nsysu.edu.tw.

1 Supplementary Figures and Tables

1.1 Supplementary Figures

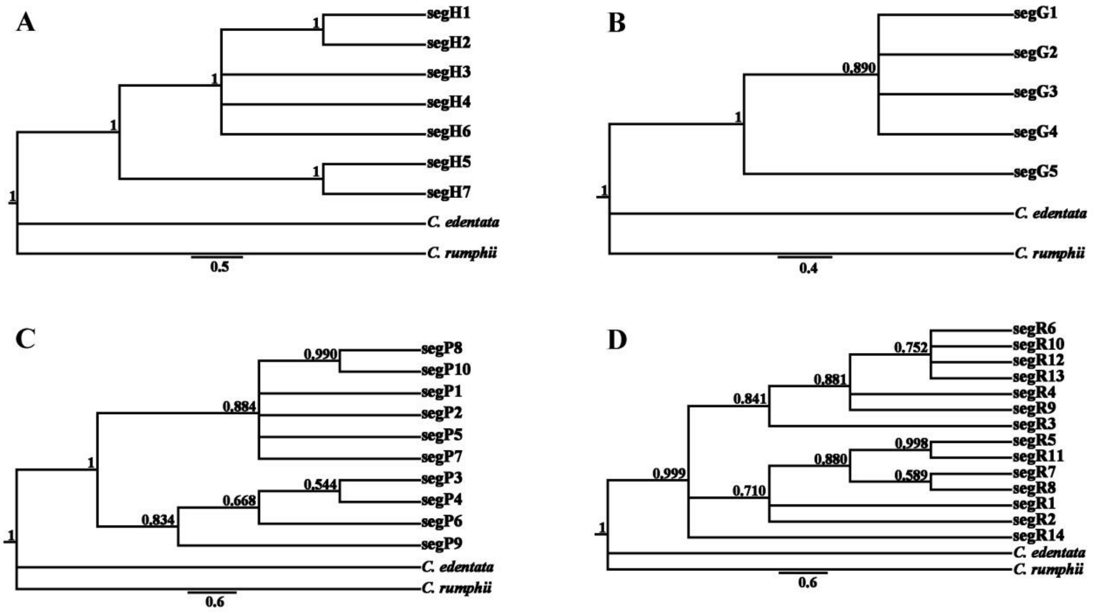

Figure S1. Bayesian tree based on the haplotypes of cpDNA (A), *GTP* (B), *PHYP* (C) and *PPRC* (D). *Cycas edentata* and *Cycas rumphii* were used as outgroups. The number on each branch indicates the posterior probability (PP).

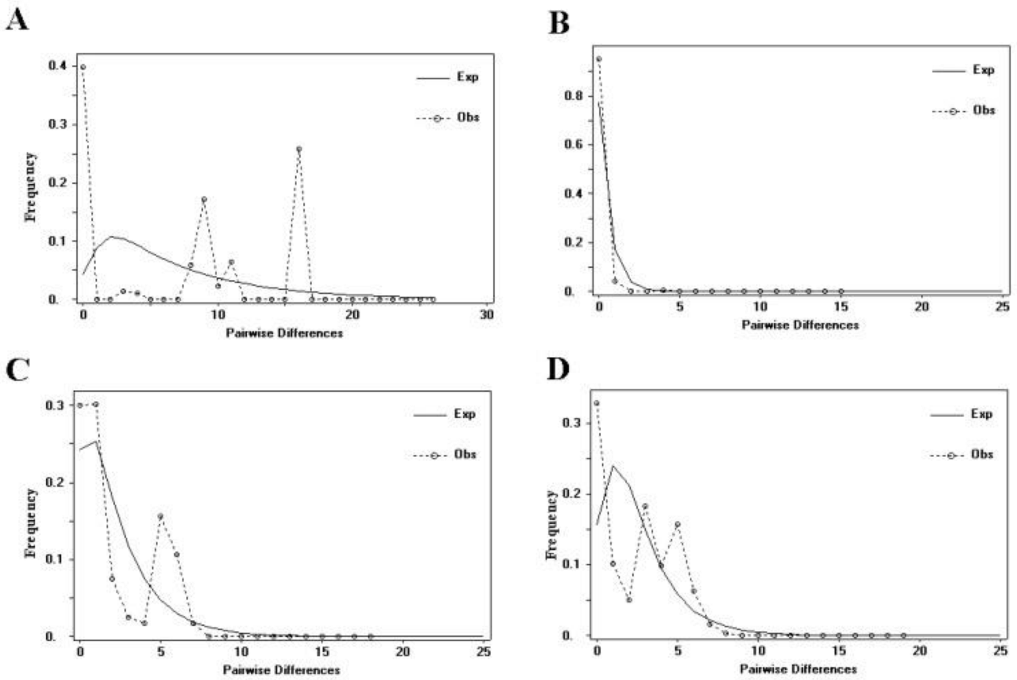

Figure S2. Mismatch distribution of cpDNA (A) and the nuclear genes *GTP* (B), *PHYP* (C) and *PPRC* (D) haplotypes based on pairwise sequence differences against the frequency of occurrence for *C. segmentifida*.

1.2 Supplementary Tables

Table S1. Details of sample locations, sample sizes surveyed for DNA sequences and microsatellites of 14 populations of *C. segmentifida*.

Population Code	Population/habitat (sand or karst)	Latitude N°	Longitude E°	Altitude m	Individuals for DNA sequences/microsatellites
BY	Bianya, Longlin, Guangxi；sand	24.754	105.468	560	10/20
JZ	Jiuzhou, Tianlin; Guangxi；sand	24.657	105.780	490	10/10
LK	Lekuan, Wangmo, Guizhou；sand	25.304	106.363	650	10/20
LKA	Lekang, Wangmo, Guizhou；sand	25.064	106.226	420	10/20
BM	Bamei, Guangnan, Yunnan；sand	24.418	104.897	960	10/20
NZ	Nazuo, Xilin, Guangxi；sand	24.148	105.393	700	10/20
BD	Badu, Tianlin, Guangxi；sand	24.327	105.827	300	10/15
BA	Boai, Funing, Yunnan；sand	23.936	106.090	300	10/20
YX	Yangxu, Baise, Guangxi；sand	23.982	106.485	350	7/7
LLB	Luolou, Lingyun, Guangxi；karst	24.367	106.810	760	10/12
SL	Shali; Lingyun, Guangxi；karst	24.241	106.811	490	10/14
PH	Pohong, Tianyang, Guangxi；karst	23.652	106.736	570	10/20
PHG	Gumei, Pohong, Tianyang, Guangxi；karst	23.605	106.643	660	10/20
BB	Bubing, Tiandong, Guangxi；karst	23.586	107.072	150	10/20
Total	14				137/238

Table S2. Haplotype diversity (*H*d) and nucleotide diversity (*P*i) estimated from cpDNA and nuclear genes as well as recombination in nuclear genes and the composition of haplotypes in *C. segmentifida*.

Population code	cpDNA (10)			*GTP* (20/0)			*PHYP* (20/1)			*PPRC* (20/2)		
	Haplotypes (No.)	*H*d	*P*i x 10^3	Haplotypes(No.) / Recombination(No.)	*H*d	*P*i x 10^3	Haplotypes(No.) / Recombination(No.)	*H*d	*P*i x 10^3	Haplotypes(No.) / Recombination(No.)	*H*d	*P*i x 10^3
BY	segH1(10)	0	0	segG1(20)	0	0	segP1(9) segP2(11)	0.521	0.56	segR1(15) segR2(5)	0.395	0.55
JZ	segH1(10)	0	0	segG1(20)	0	0	segP1(7) segP2(10) segP3(3)	0.637	2.30	segR2(19) segR3(1)	0.100	0.84
LK	segH1(10)	0	0	segG1(20)	0	0	segP1(15) segP4(5)	0.395	2.12	segR2(20)	0	0
LKA	segH1(10)	0	0	segG1(20)	0	0	segP1(4) segP4(16)	0.337	1.81	segR2(20)	0	0
BM	segH1(10)	0	0	segG1(20)	0	0	segP1(5) segP2(15)	0.395	0.42	segR2(20)	0	0
NZ	segH2(10)	0	0	segG1(20)	0	0	segP1(2) segP2(11) segP5(7)	0.595	1.08	segR1(4) segR2(15) segR5(1)	0.416	1.17
BD	segH1(10)	0	0	segG1(20)	0	0	segP1(12) segP2(3) segP4(3) segP5(2)	0.616	1.94	segR2(20)	0	0
BA	segH1(10)	0	0	segG1(20)	0	0	segP1(15) segP5(5)	0.395	0.42	segR2(7) segR4(13)	0.479	2.00
YX	segH3(7)	0	0	segG1(13) segG2(1)	0.143	0.25	segP1(9) segP2(3) segP6(2)	0.560	1.52	segR2(4) segR4(4) segR5(2) segR6(3) segR7(1)	0.824	4.44
LLB	segH4(10)	0	0	segG1(20)	0	0	segP1(13) segP2(1) segP4(2) segP5(4)	0.553	1.49	segR4(1) segR5(11) segR7(8)	0.563	2.71
SL	segH5(10)	0	0	segG1(20)	0	0	segP1(13) segP5(1) segP7(6)	0.511	1.06	segR4(10) segR5(9) segR6(1)	0.574	4.49
PH	segH6(10)	0	0	segG1(19) segG3(1)	0.100	0.18	segP1(8) segP2(3) segP4(6) segP7(2) segP8(1)	0.753	3.18	segR2(6) segR4(3) segR5(4) segR6(5) segR7(1) segR8(1)	0.821	4.97
PHG	segH5(10)	0	0	segG1(19) segG2(1)	0.100	0.18	segP1(11) segP2(2) segP4(2) segP5(2) segP9(3)	0.679	2.31	segR2(5) segR4(3) segR5(2) segR6(3) segR7(1) segR9-segR14(1)	0.911	4.79
BB	segH5(6) segH7(4)	0.533	1.7	segG1(16) segG3(1) segG4(2) segG5(1)	0.363	1.23	segP1(7) segP4(8) segP8(4) segP10(1)	0.711	3.25	segR2(5) segR4(8) segR5(1) segR6(5) segR12(1)	0.747	3.09
Total	7	0.602	2.29	5	0.05	0.13	10	0.700	2.31	14	0.673	3.42

Table S3. Genetic diversity, differentiation parameters based on the combined cpDNA sequences and nuclear genes in *C. segmentifida*.

Marker	H_S	H_T	G_{ST}	N_{ST}
cpDNA	0.038	0.745	0.949	0.996
GTP	0.050	0.053	0.040	0.008
PHYP	0.547	0.709	0.229	0.282
PPRC	0.416	0.696	0.402	0.415

Table S4. Parameters of neutrality tests and mismatch analysis based on cpDNA and nuclear genes of *C. segmentifida*.

Marker	Tajima'*D*	Fu and Li'*D**	Fu and Li'*F**	Fu'*F*s	SSD	raggedness
cpDNA	2.45969*	1.77508**	2.44516**	17.413***	0.00217*	0.02063*
GTP	-1.91209*	-2.81556*	-2.98755**	-6.974***	0.00088	0.14636
PHYP	0.24935	0.68737	0.62868	0.763	0.09708	0.26289
PPRC	-0.12793	-1.47618	-1.14551	-0.791	0.05944	0.23616

Note: *, $P < 0.05$, significant difference; **, $P < 0.01$, most significant difference; ***, $P < 0.001$, most significant difference.

Table S5. Summary of the twelve microsatellite loci used to study the population genetics among 11 populations of *C. segmentifida*.

Locus	A_R	N_A	A_E	I	H_O	H_E	F_{IS}	F_{IT}	F_{ST}	H_S	H_T	G_{ST}	G'_{ST}	D_{est}
Cha02	3.824	14	4.985	1.924	0.584	0.801	0.111	0.284	0.194	0.680	0.814	0.165	0.175	0.134
Cha08	4.061	20	5.190	2.250	0.397	0.809	0.295	0.518	0.317	0.597	0.836	0.285	0.301	0.238
Cy-TaiEST-SSR11	1.159	3	1.047	0.117	0.046	0.045	-0.084	-0.018	0.060	0.037	0.039	0.032	0.035	0.001
E001	1.195	5	1.908	0.910	0.294	0.477	-0.070	0.358	0.440	0.277	0.448	0.382	0.399	0.171
E004	3.982	10	5.122	1.857	0.702	0.806	-0.020	0.146	0.163	0.703	0.814	0.136	0.145	0.111
Cpz26	2.274	8	2.182	0.988	0.391	0.543	-0.027	0.259	0.279	0.393	0.529	0.256	0.270	0.135
HL08	2.200	9	1.901	1.011	0.332	0.475	-0.085	0.328	0.381	0.312	0.490	0.362	0.380	0.177
CY232	1.937	4	1.529	0.614	0.265	0.347	0.013	0.211	0.201	0.292	0.354	0.175	0.186	0.062
Cha-estssr01	5.480	44	17.624	3.189	0.340	0.945	0.526	0.614	0.187	0.808	0.946	0.146	0.156	0.138
Cha-estssr02	2.761	6	2.840	1.203	0.479	0.649	0.015	0.223	0.211	0.532	0.652	0.185	0.196	0.120
Cha-estssr04	4.217	13	7.463	2.135	0.710	0.868	-0.031	0.173	0.197	0.721	0.870	0.172	0.183	0.149
Cha05	1.301	4	1.102	0.216	0.088	0.093	-0.090	0.031	0.111	0.086	0.094	0.084	0.090	0.008
Mean	2.929	11.667	4.408	1.368	0.386	0.572	0.046	0.261	0.225	0.453	0.574	0.210	0.223	0.120
Total		117												

Table S6. *P*-value of Hardy-Weinberg equilibrium test for 14 populations of *C. segmentifida*.

Population	Cha02	Cha08	Cy-TaiEST-SSR11	E001	E004	Cpz26	HL08	CY232	Cha-estssr01	Cha-estssr02	Cha-estssr04	Cha05	All loci
BY	0.004**	0.006**	-	1.000^{ns}	0.324^{ns}	0.006**	-	1.000^{ns}	0.000***	0.049*	0.021*	-	0.000***
JZ	0.019*	0.000***	-	1.000^{ns}	0.152^{ns}	0.184^{ns}	-	1.000^{ns}	0.484^{ns}	0.101^{ns}	0.462^{ns}	1.000^{ns}	0.005**
LK	0.054^{ns}	-	-	1.000^{ns}	0.036*	0.707^{ns}	-	-	0.000***	0.001**	1.000^{ns}	-	0.000***
LKA	0.202^{ns}	1.000^{ns}	-	0.591^{ns}	0.069*	0.468^{ns}	-	-	0.000***	0.021*	0.913^{ns}	0.026*	0.000***
BM	0.880^{ns}	1.000^{ns}	-	0.277^{ns}	0.112^{ns}	1.000^{ns}	-	-	0.043*	1.000^{ns}	0.800^{ns}	1.000^{ns}	0.733^{ns}
NZ	0.001**	0.000***	-	1.000^{ns}	0.056^{ns}	-	1.000^{ns}	0.174^{ns}	0.028*	0.002**	0.017*	1.000^{ns}	0.000***
BD	0.265^{ns}	0.000***	-	0.652^{ns}	0.151^{ns}	0.528^{ns}	1.000^{ns}	0.628^{ns}	0.017*	0.775^{ns}	0.970^{ns}	-	0.000***
BA	1.000^{ns}	0.000***	-	-	0.522^{ns}	1.000^{ns}	0.350^{ns}	1.000^{ns}	0.000***	0.257^{ns}	0.983^{ns}	-	0.000***
YX	0.007**	0.009**	-	-	0.100^{ns}	1.000^{ns}	1.000^{ns}	0.441^{ns}	0.009**	1.000^{ns}	0.359^{ns}	-	0.005**
LLB	0.123^{ns}	0.976^{ns}	-	0.529^{ns}	1.000^{ns}	1.000^{ns}	1.000^{ns}	1.000^{ns}	0.027*	1.000^{ns}	0.894^{ns}	-	0.880^{ns}
SL	1.000^{ns}	0.561^{ns}	-	-	0.138^{ns}	0.238^{ns}	1.000^{ns}	0.150^{ns}	0.000***	0.348^{ns}	0.308^{ns}	-	0.000***
PH	0.124^{ns}	0.000***	1.000^{ns}	0.145^{ns}	0.705^{ns}	0.754^{ns}	0.613^{ns}	0.468^{ns}	0.000***	1.000^{ns}	0.698^{ns}	-	0.000***
PHG	0.040*	0.009**	1.000^{ns}	0.543^{ns}	0.176^{ns}	0.657^{ns}	0.728^{ns}	1.000^{ns}	0.000***	1.000^{ns}	0.239^{ns}	-	0.000***
BB	0.763^{ns}	0.903^{ns}	1.000^{ns}	0.242^{ns}	0.241^{ns}	1.000^{ns}	0.732^{ns}	1.000^{ns}	0.000***	0.480^{ns}	0.996^{ns}	-	0.000***
All pop.	0.000***	0.000***	1.000^{ns}	0.913^{ns}	0.009**	0.718^{ns}	0.972^{ns}	0.969^{ns}	0.000***	0.005**	0.551^{ns}	0.502^{ns}	0.000***

Note: -, Monomorphic; ns, non-significance; *, $P < 0.05$, significant difference; **, $P < 0.01$, most significant difference; ***, $P < 0.001$, most significant difference.
